# Supplementary material for: Intermittent light enhances pigment production in the diatom Phaeodactylum tricornutum: a combined physiological and transcriptomic approach
Source: Microbiol Spectr. 2026 May 14;14(6):e03449-25. doi: 10.1128/spectrum.03449-25 (PMC13227974; doi:10.1128/spectrum.03449-25)
Supplement: Supplemental material — Fig. S1 and S2, Tables S1 to S3. [file spectrum.03449-25-s0001.docx]

**Supplemental Materials：**


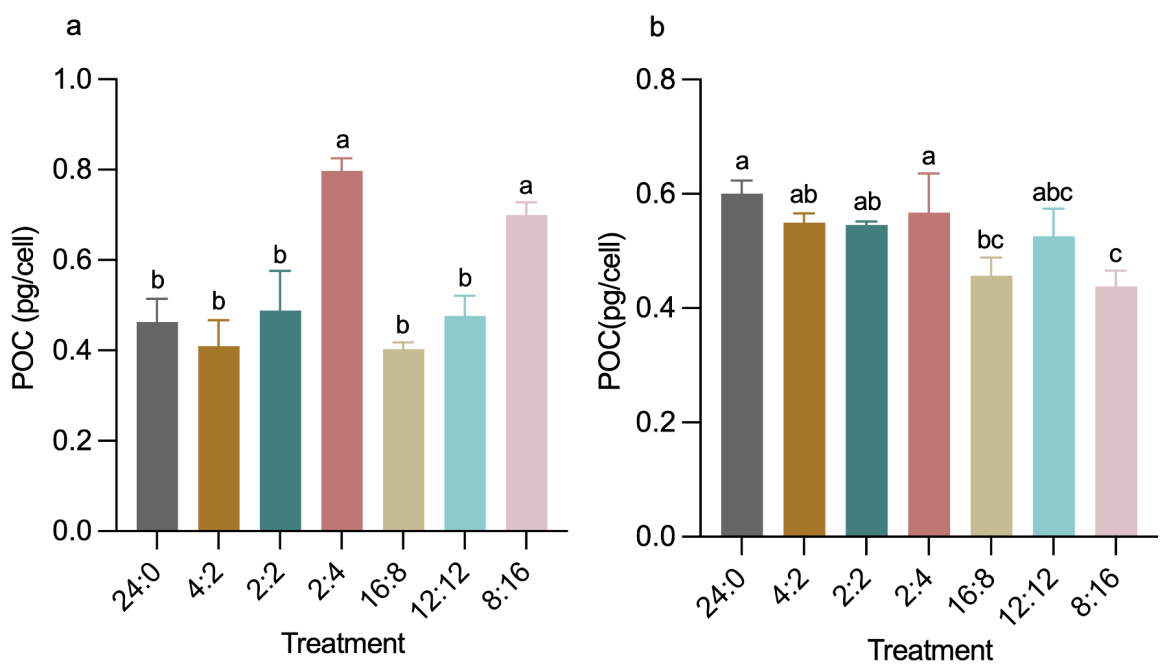

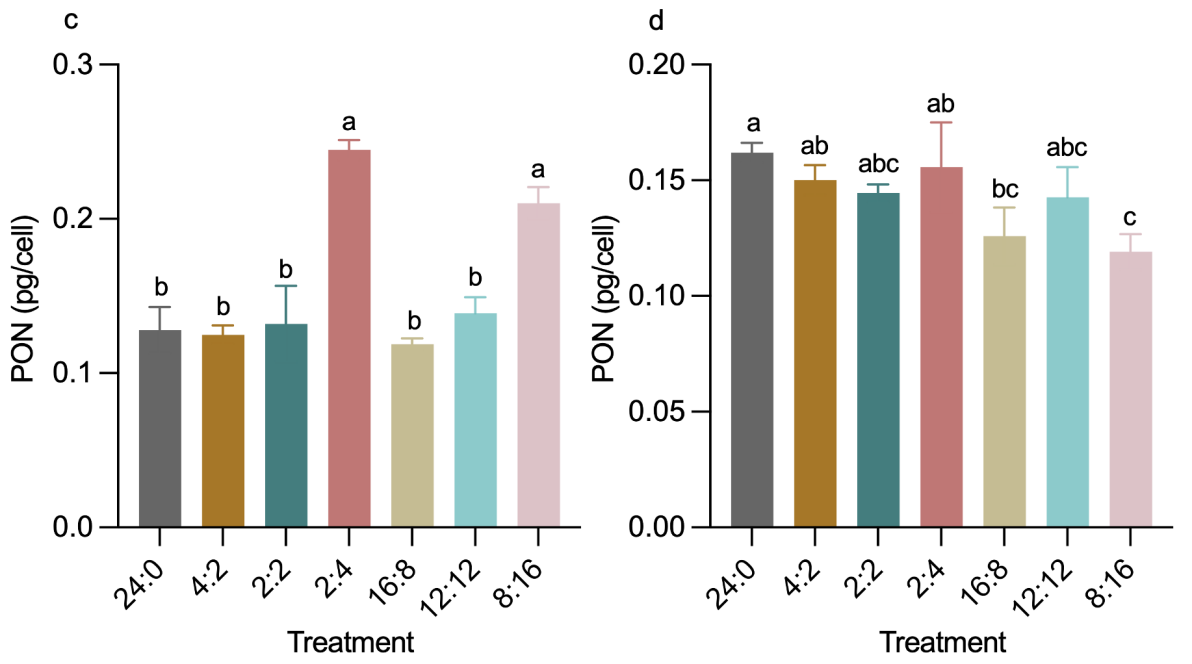


**
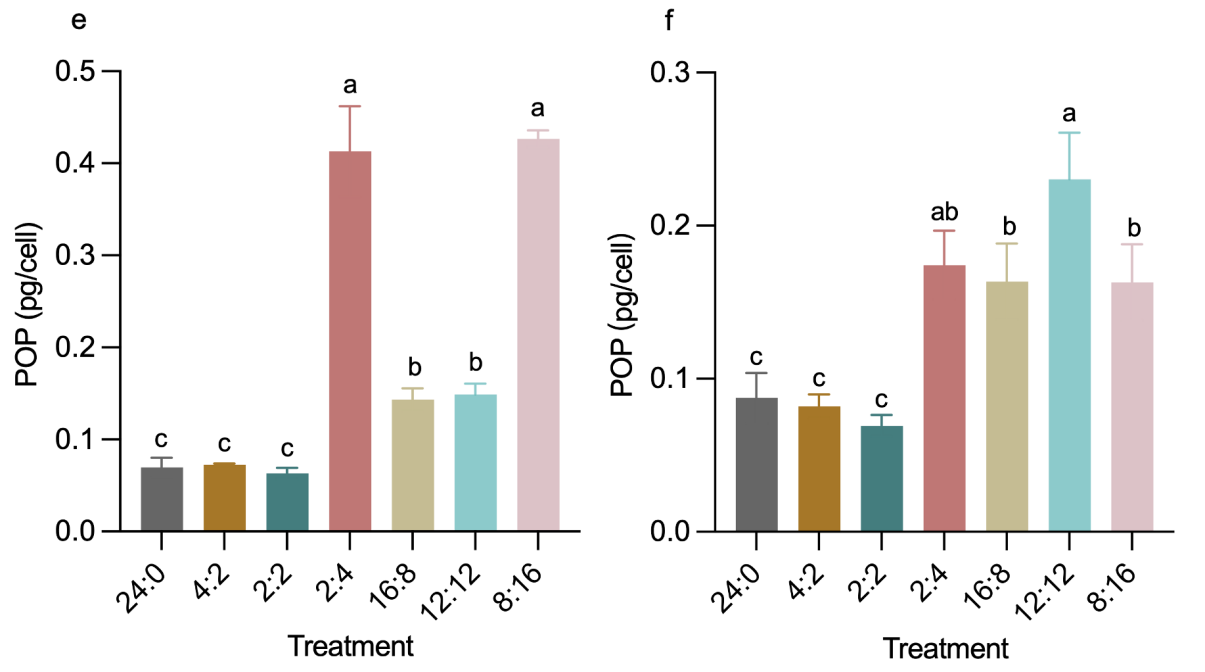
**


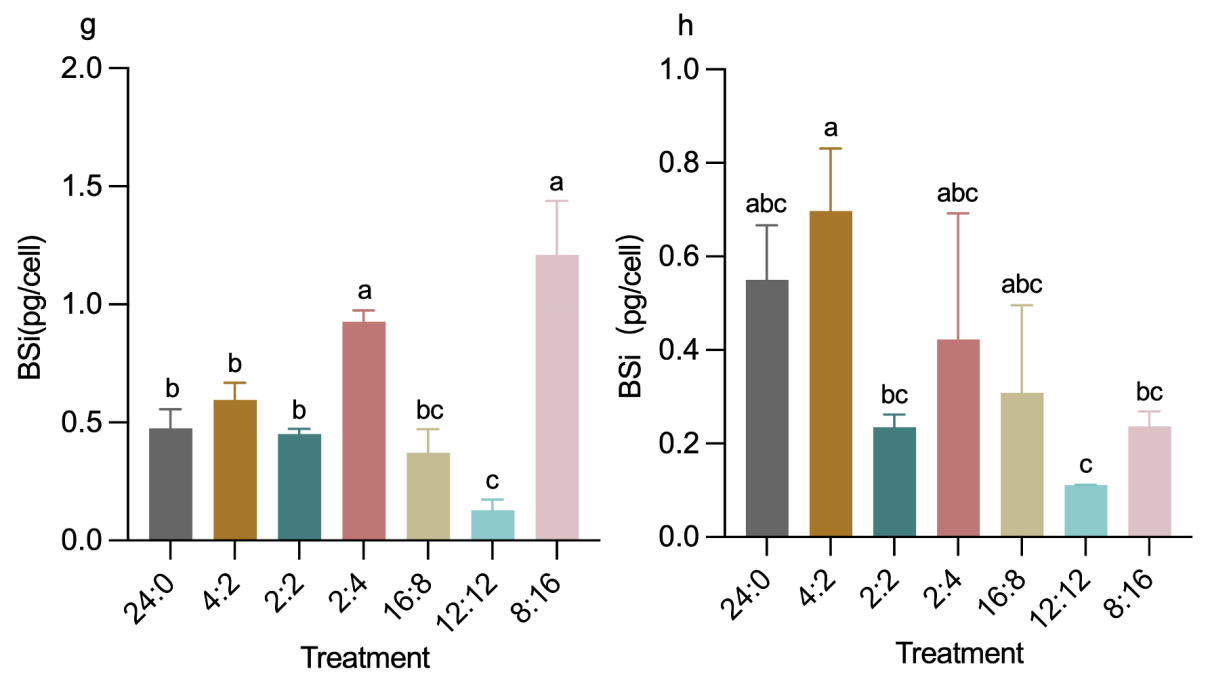


Figure S1: Intracellular elemental contents under different light-dark cycles (a) Intracellular POC at exponential phase; (b) Intracellular POC at stationary phase; (c) Intracellular PON at exponential phase; (d) Intracellular PON at stationary phase; (e) Intracellular POP at exponential phase; (f) Intracellular POP at stationary phase; (g) Intracellular BSi at exponential phase and (h) Intracellular BSi at stationary phase. Data from day 6 represent the exponential phase, and day 10 represents the stationary phase. Error bars indicate standard deviation (n = 3). Different letters indicate significant differences between treatments (*p* < 0.05).

**
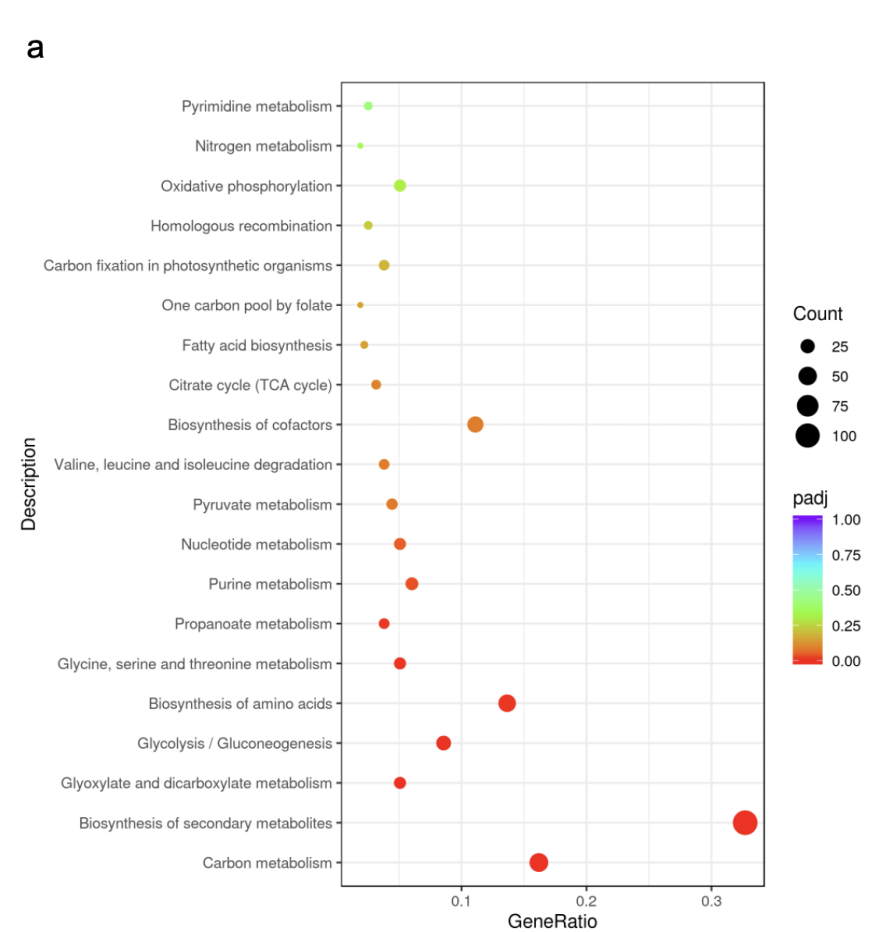

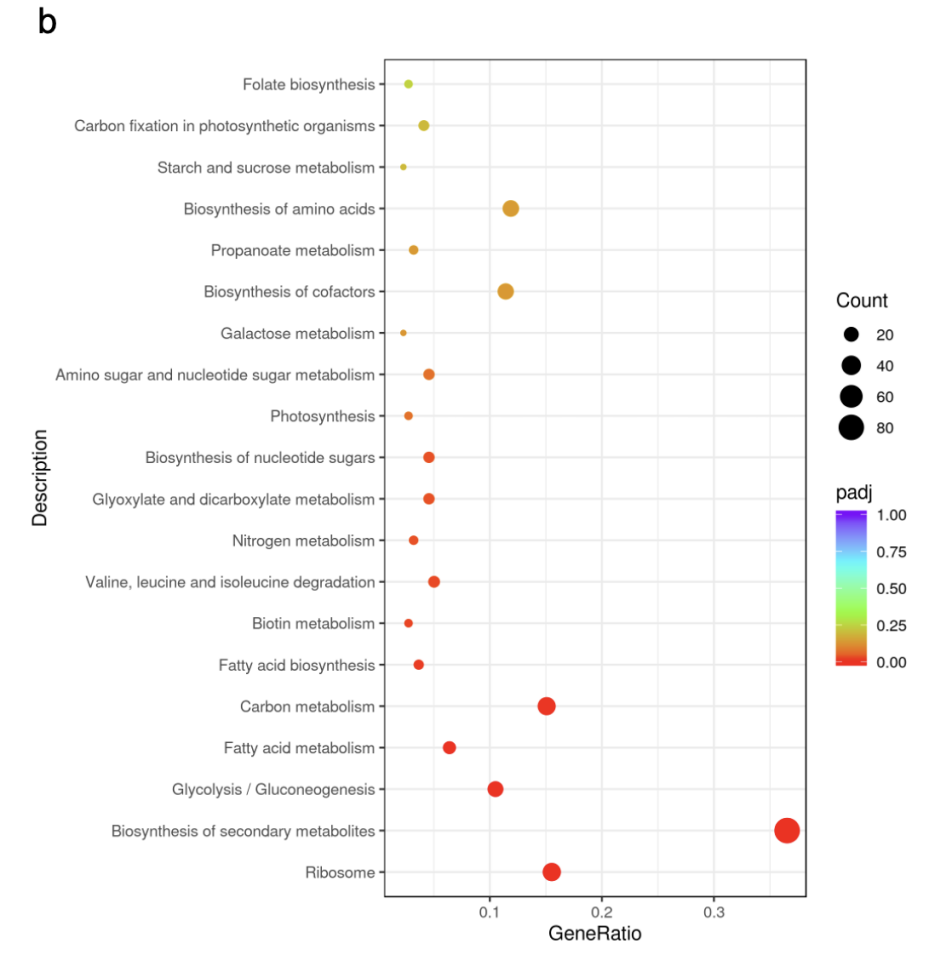
**

**
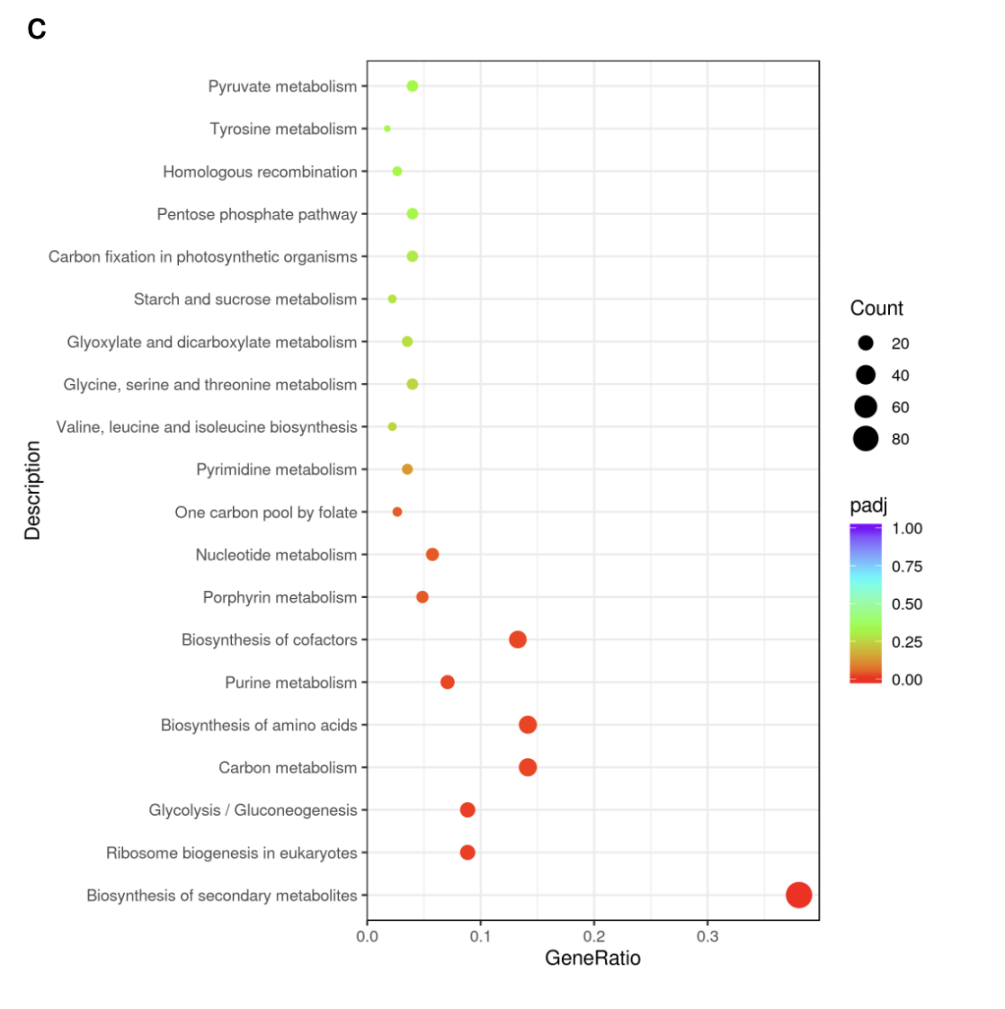
**

Figure S2: KEGG enrichment analysis dot plot. (a. LD 2:2 vs LD 12:12, b. LD 2:2 vs LD 24:0, c. LD 12:12 vs LD 24:0)

Table S1: Statistics of differentially expressed genes (DEGs) related to pigment biosynthesis by comparison between LD 2:2 and LD 12:12.

| **gene name** | **Expression in LD 2:2 (FPKM)** | | | **Expression in LD 12:12 (FPKM)** | | | **\|log_2_foldchange\|** | **padj** |
| --- | --- | --- | --- | --- | --- | --- | --- | --- |
|  | **LD2:2-1** | **LD2:2-2** | **LD2:2-3** | **LD12:2-1** | **LD12:12-2** | **LD12:12-3** |  |  |
| Lhcr10 | 319.23 | 252.86 | 282.90 | 21.55 | 29.42 | 26.54 | 3.47 | 5.90E-59 |
| Lhcr6 | 452.65 | 374.24 | 418.91 | 133.49 | 152.87 | 95.27 | 1.72 | 1.18E-12 |
| Lhcr7 | 5.89 | 6.55 | 6.85 | 18.68 | 12.30 | 16.36 | -1.28 | 1.70E-05 |
| Lhcf3 | 679.33 | 614.15 | 677.30 | 2479.31 | 909.84 | 1209.14 | -1.21 | 1.43E-03 |
| Lhcf5 | 2998.03 | 2597.40 | 2548.20 | 17095.52 | 5161.38 | 7098.10 | -1.83 | 9.99E-06 |
| Lhcf10 | 2872.01 | 2168.75 | 1689.75 | 6190.30 | 2967.67 | 4828.15 | -1.04 | 2.42E-03 |
| PHATRDRAFT_48798 | 475.54 | 400.49 | 326.11 | 1423.38 | 683.00 | 911.50 | -1.31 | 4.08E-05 |
| Lhcf16 | 174.57 | 140.45 | 126.27 | 91.25 | 33.12 | 27.47 | 1.56 | 6.08E-04 |
| Lhcx1 | 2834.09 | 2456.32 | 2068.68 | 610.34 | 134.02 | 106.61 | 3.13 | 8.83E-04 |
| PHATRDRAFT_43374 | 20.09 | 17.26 | 17.29 | 1.81 | 2.76 | 3.66 | 2.74 | 2.13E-19 |
| PHATRDRAFT_37960 | 18.86 | 21.53 | 21.35 | 43.93 | 45.61 | 56.87 | -1.23 | 2.03E-08 |
| VDL2 | 46.71 | 44.12 | 40.53 | 121.14 | 101.95 | 109.44 | -1.33 | 4.58E-13 |
| PHATRDRAFT_56588 | 186.77 | 159.10 | 86.04 | 873.27 | 468.73 | 550.37 | -2.12 | 1.89E-09 |
| Dph1 | 5.77 | 7.34 | 7.22 | 9.50 | 15.69 | 17.58 | -1.06 | 7.98E-04 |
| PDS-like1 | 13.57 | 12.30 | 21.25 | 4.36 | 6.40 | 9.78 | 1.21 | 2.80E-03 |

Table S2: Statistics of differentially expressed genes (DEGs) related to pigment biosynthesis by comparison between LD 2:2 and LD 24:0.

| **gene name** | **Expression in LD 2:2 (FPKM)** | | | **Expression in LD 24:0 (FPKM)** | | | **\|log_2_foldchange\|** | **padj** |
| --- | --- | --- | --- | --- | --- | --- | --- | --- |
|  | **LD2:2-1** | **LD2:2-2** | **LD2:2-3** | **LD24:0-1** | **LD24:0-2** | **LD24:0-3** |  |  |
| Lhcr7 | 5.89 | 6.55 | 6.85 | 21.76 | 19.85 | 19.42 | -1.68 | 1.84E-16 |
| Lhcr8 | 14.88 | 14.06 | 34.79 | 54.64 | 64.14 | 40.81 | -1.34 | 2.88E-04 |
| Lhcr13 | 1994.10 | 1676.87 | 1769.02 | 900.73 | 728.22 | 571.61 | 1.28 | 1.42E-10 |
| PHATRDRAFT_24119 | 161.67 | 136.79 | 135.48 | 85.36 | 73.74 | 40.64 | 1.10 | 6.44E-05 |
| PHATRDRAFT_17531 | 1669.09 | 1419.99 | 1325.11 | 749.94 | 663.64 | 601.68 | 1.11 | 2.17E-11 |
| Lhcf17 | 189.30 | 152.87 | 152.42 | 96.98 | 68.56 | 56.19 | 1.14 | 1.69E-06 |
| Lhcf3 | 679.33 | 614.15 | 677.30 | 110.58 | 98.74 | 89.09 | 2.70 | 8.26E-76 |
| Lhcf4 | 1682.32 | 1388.80 | 1179.46 | 394.85 | 311.29 | 265.78 | 2.11 | 1.07E-24 |
| Lhcf8 | 742.81 | 608.72 | 574.89 | 223.34 | 213.17 | 155.52 | 1.68 | 8.08E-18 |
| Lhcf11 | 1342.96 | 1172.21 | 1106.59 | 2986.84 | 2445.60 | 2424.94 | -1.14 | 1.95E-12 |
| Lhcf16 | 174.57 | 140.45 | 126.27 | 41.38 | 40.32 | 38.05 | 1.86 | 1.14E-24 |
| Lhcx1 | 2834.09 | 2456.32 | 2068.68 | 582.28 | 663.04 | 337.06 | 2.20 | 5.36E-18 |
| PHATRDRAFT_43374 | 20.09 | 17.26 | 17.29 | 8.55 | 8.52 | 8.22 | 1.09 | 4.61E-10 |
| PHATRDRAFT_37960 | 18.86 | 21.53 | 21.35 | 63.62 | 66.76 | 62.13 | -1.66 | 5.95E-29 |
| PHATRDRAFT_56588 | 186.77 | 159.10 | 86.04 | 572.06 | 552.56 | 525.71 | -1.96 | 6.38E-14 |
| PHATRDRAFT_46085 | 50.09 | 40.39 | 33.32 | 15.69 | 12.13 | 8.50 | 1.75 | 4.96E-11 |
| PHATR_43904 | 7.06 | 6.42 | 5.97 | 18.70 | 15.11 | 19.79 | -1.48 | 2.55E-14 |
| ZEP1 | 29.04 | 27.65 | 18.95 | 11.65 | 11.60 | 7.36 | 1.28 | 1.28E-06 |
| ZEP3 | 145.25 | 102.32 | 70.89 | 50.51 | 56.23 | 30.14 | 1.20 | 1.29E-04 |

Table S3: Statistics of differentially expressed genes (DEGs) related to pigment biosynthesis by comparison between LD 12:12 and LD 24:0.

| **gene name** | **Expression in LD 12:12 (FPKM)** | | | **Expression in LD 24:0 (FPKM)** | | | **\|log_2_foldchange\|** | **padj** |
| --- | --- | --- | --- | --- | --- | --- | --- | --- |
|  | **LD12:2-1** | **LD12:12-2** | **LD12:12-3** | **LD24:0-1** | **LD24:0-2** | **LD24:0-3** |  |  |
| Lhcr1 | 2294.16 | 989.64 | 1415.20 | 696.78 | 697.69 | 571.40 | 1.26 | 1.70E-04 |
| Lhcr6 | 133.49 | 152.87 | 95.27 | 286.35 | 285.56 | 205.42 | -1.02 | 2.62E-04 |
| Lhcr8 | 26.13 | 21.52 | 5.10 | 54.64 | 64.14 | 40.81 | -1.59 | 3.41E-03 |
| Lhcr10 | 21.55 | 29.42 | 26.54 | 654.71 | 514.99 | 321.76 | -4.26 | 3.17E-47 |
| Lhcr11 | 1802.00 | 694.33 | 804.94 | 387.69 | 341.17 | 273.80 | 1.72 | 7.36E-06 |
| Lhcr13 | 2674.78 | 1235.14 | 1328.75 | 900.73 | 728.22 | 571.61 | 1.25 | 5.86E-04 |
| Lhcf2 | 3367.88 | 1585.72 | 2324.49 | 1342.46 | 1153.05 | 1093.84 | 1.02 | 1.23E-03 |
| Lhcf12 | 127.41 | 71.46 | 92.51 | 41.39 | 33.57 | 22.53 | 1.58 | 2.81E-06 |
| Lhcf3 | 2479.31 | 909.84 | 1209.14 | 110.58 | 98.74 | 89.09 | 3.95 | 5.91E-29 |
| Lhcf4 | 4161.90 | 1348.30 | 1988.32 | 394.85 | 311.29 | 265.78 | 2.95 | 5.45E-14 |
| Lhcf5 | 17095.52 | 5161.38 | 7098.10 | 1558.91 | 1291.59 | 1773.43 | 2.67 | 9.85E-11 |
| Lhcf8 | 1382.30 | 623.81 | 905.12 | 223.34 | 213.17 | 155.52 | 2.30 | 7.06E-13 |
| Lhcf9 | 1567.45 | 475.63 | 754.48 | 447.29 | 420.91 | 336.76 | 1.22 | 4.77E-03 |
| Lhcf10 | 6190.30 | 2967.67 | 4828.15 | 2132.03 | 2292.46 | 2256.98 | 1.07 | 4.13E-04 |
| PHATRDRAFT_48798 | 1423.38 | 683.00 | 911.50 | 242.81 | 256.41 | 216.67 | 2.08 | 1.45E-12 |
| PHATRDRAFT_47485 | 1344.73 | 392.35 | 441.79 | 391.26 | 366.46 | 299.34 | 1.05 | 3.27E-02 |
| Lhcx3 | 10.32 | 10.21 | 6.03 | 23.72 | 28.26 | 26.16 | -1.55 | 5.21E-07 |
| VDL2 | 121.14 | 101.95 | 109.44 | 25.17 | 22.99 | 22.43 | 2.24 | 2.23E-37 |
| PHATRDRAFT_43374 | 1.81 | 2.76 | 3.66 | 8.55 | 8.52 | 8.22 | -1.61 | 1.98E-06 |
| PSY | 100.47 | 104.90 | 98.29 | 42.32 | 42.89 | 26.06 | 1.45 | 1.91E-08 |
| PHATR_43904 | 8.60 | 4.55 | 5.91 | 18.70 | 15.11 | 19.79 | -1.49 | 1.52E-06 |
| CRTISO5 | 122.87 | 84.89 | 99.52 | 48.88 | 66.24 | 38.26 | 1.01 | 6.46E-04 |
| CRTISO1 | 12.32 | 9.77 | 10.21 | 5.94 | 5.66 | 3.37 | 1.11 | 7.30E-04 |
| PHATRDRAFT_44908 | 62.87 | 39.68 | 44.39 | 24.31 | 21.99 | 15.18 | 1.26 | 2.01E-05 |
| CPF1 | 26.29 | 32.38 | 30.59 | 71.16 | 71.15 | 48.05 | -1.09 | 2.04E-05 |
| ZEP1 | 42.80 | 17.62 | 18.24 | 11.65 | 11.60 | 7.36 | 1.36 | 1.41E-03 |
